# Association between haemoglobin level and anthropometric status of children under 5 years receiving health care services at Parklane Teaching Hospital, Enugu

**DOI:** 10.3389/fmed.2026.1969062

**Published:** 2026-10-14

**Authors:** Emmanuel Ifeanyi Obeagu, Nwanganga Ihuoma Ubosi

**Affiliations:** 1 Department of Biomedical and Laboratory Science, Africa University, Mutare, Zimbabwe; 2 Department of Molecular Medicine and Haematology, Faculty of Health Sciences, University of the Witwatersrand, Johannesburg, South Africa; 3 Department of Haematology and Immunohaematology, Bahir Dar University, Bahir Dar, Ethiopia; 4 Department of Public Health, National Open University, Abuja, Nigeria

**Keywords:** anaemia, anthropometry, haemoglobin level, socioeconomic status, under-five children

## Abstract

**Introduction:**

Anaemia remains an important public health concern among children under five years in sub-Saharan Africa, with nutritional, socioeconomic, and other environmental factors potentially contributing to impaired haemoglobin status and growth. However, the relationship between haemoglobin status and anthropometric characteristics among young children in Nigeria remains insufficiently characterized. This study assessed the association between haemoglobin concentration and anthropometric status among children under five years receiving healthcare services at Parklane Teaching Hospital, Enugu.

**Methods:**

A cross-sectional study was conducted among 384 children aged 0–59 months. Data were obtained using structured questionnaires, anthropometric measurements, and laboratory assessment of haemoglobin concentration and packed cell volume (PCV). Maternal nutritional characteristics, socioeconomic status, parental occupation, income, and other relevant demographic variables were assessed. Weight-for-age, length/height-for-age, weight-for-length/height, and BMI-for-age Z-scores were determined using WHO Child Growth Standards. Associations between haemoglobin status, anthropometric indicators, and selected demographic and socioeconomic characteristics were assessed using appropriate statistical tests, with statistical significance set at *p* < 0.05.

**Results:**

Weight-for-age was significantly associated with haemoglobin status, maternal nutritional characteristics, socioeconomic status, parental income, and occupation (*p* < 0.001 for the respective significant associations), but not with PCV. Length/height-for-age was significantly associated with haemoglobin concentration (*p* < 0.001), PCV (*p* = 0.002), and selected socioeconomic characteristics. BMI-for-age was significantly associated with haemoglobin concentration (*p* < 0.013), PCV (*p* < 0.001), maternal nutritional characteristics (*p* < 0.002), and parental occupation (*p* < 0.032). Weight-for-length/height was not significantly associated with haemoglobin status or most socioeconomic characteristics but was associated with PCV and maternal nutritional characteristics (*p* < 0.001). Age, sex, parental education, income, and maternal nutritional characteristics also demonstrated significant associations with haemoglobin status.

**Conclusion:**

Haemoglobin status was associated with several anthropometric and socioeconomic characteristics among children under five years attending the study facility. The findings support an integrated approach to child health involving haemoglobin assessment, growth monitoring, nutritional evaluation, and appropriate counselling of children and their caregivers. Broader preventive strategies should also address maternal and household factors that may influence childhood nutrition and growth. Because of the cross-sectional design, these associations should not be interpreted as causal.

## Introduction

Anaemia and malnutrition rank among the top causes of childhood illness and death globally, especially in low- and middle-income countries (LMICs). Worldwide, anaemia impacts roughly 42% of children under five, with nutritional shortages, infections, and genetic conditions playing a role in its significant occurrence. Malnutrition, marked by stunting, wasting, and low weight, continues to be a concurrent issue that threatens child health, growth, and survival. These paired conditions frequently exist together and influence each other, resulting in a detrimental cycle of weakened immunity, inadequate growth, and delays in development (1, 2). The prevalence of anaemia and malnutrition is disproportionately elevated in sub-Saharan Africa, where food scarcity, restricted healthcare access, and elevated rates of infectious diseases worsen the issue. In Nigeria, the prevalence of childhood anaemia is still worryingly high, varying between 50% and 70% based on the region and socio-economic factors. Malnutrition is also prevalent, with stunting impacting almost one-third of children in Nigeria who are under five years old. These factors together represent a considerable share of avoidable fatalities and lasting impairments in children (3, 4).

Haemoglobin levels serve as a dependable and commonly utilized indicator for evaluating anaemia in young patients. Low hemoglobin levels indicate insufficient oxygen transport in the blood, which undermines energy metabolism, cognitive growth, and immune function. Conversely, anthropometric metrics like weight-for-age, height-for-age, and weight-for-height z-scores serve as crucial indicators of nutritional health. Collectively, these parameters offer important information regarding the growth and well-being of children and act as essential tools for clinical evaluation and public health observation. The relationship between anaemia and malnutrition is well established, but it is still not thoroughly investigated in numerous situations. Inadequate intake of iron and other micronutrients can lead to anaemia, which in turn can exacerbate growth retardation by limiting oxygen supply to tissues vital for growth. Infections like malaria and helminthiasis complicate this connection by leading to nutrient depletion and haemolysis. Grasping these interactions is crucial for creating combined interventions that address anaemia and malnutrition at the same time (5).

Although many studies have been carried out throughout Africa, there is a lack of research in southeastern Nigeria exploring the direct relationship between haemoglobin levels and the anthropometric status of children under five. Many existing studies have concentrated exclusively on the prevalence of anaemia or nutritional indices separately, without exploring their connections within clinical populations. This results in a lack of understanding that obstructs the creation of evidence-informed approaches designed to meet the needs of children utilizing healthcare services in this area (6, 7). Parklane Teaching Hospital in Enugu functions as a key referral hub for pediatric services in southeastern Nigeria and offers an excellent environment for these studies. The hospital serves various socio-economic categories, providing a chance to collect data that represent the multiple factors influencing child health in the area. Examining the relationship between haemoglobin levels and anthropometric measures in this context can enhance both hospital clinical practices and broader public health strategies. This research seeks to evaluate the relationship between haemoglobin levels and anthropometric measurements in children younger than five years visiting Parklane Teaching Hospital, Enugu. By connecting haematological and nutritional factors, the research aims to enhance the comprehension of how these interconnected conditions appear in a Nigerian child population. The results may offer support for combined strategies addressing both anaemia and malnutrition, thus enhancing child health results in Nigeria and comparable LMIC environments.

This study aimed to determine the association between haemoglobin level and anthropometric status among children aged 0–59 months receiving healthcare services at Parklane Teaching Hospital, Enugu. Specifically, the study assessed haemoglobin levels and the prevalence of anaemia, evaluated anthropometric status using WHO growth indicators, determined the association between haemoglobin/anaemia status and anthropometric measures, and examined selected sociodemographic and maternal characteristics in relation to these outcomes. The primary outcome was the association between haemoglobin level or anaemia status and anthropometric status, while prevalence estimates and selected sociodemographic and maternal characteristics were considered secondary outcomes.

## Methodology

### Study design

This study employed a hospital-based cross-sectional analytical design to determine haemoglobin levels and anthropometric status and to assess their association among children aged 0–59 months receiving healthcare services at Parklane Teaching Hospital, Enugu, Nigeria. The cross-sectional design enabled the simultaneous assessment of haemoglobin and anthropometric measurements at a defined period, providing estimates of anaemia and nutritional status and allowing examination of associations between these variables. Because exposure and outcome variables were assessed at the same time, the study does not establish temporal or causal relationships.

### Study area

The research took place at Parklane Teaching Hospital, a tertiary healthcare facility situated in Enugu, Nigeria. This hospital caters to a significant population and acts as a main referral center for pediatric services in the area. Location and Geography Parklane Teaching Hospital is situated in Enugu, the capital of Enugu State in the southeastern region of Nigeria. Enugu, referred to as the “Coal City” for its abundant coal reserves, is located at an altitude of approximately 223 meters above sea level. The city has a tropical savanna climate characterized by two distinct seasons: the wet season (April to October) and the dry season (November to March). Enugu is an active metropolitan area with a population believed to exceed 700,000 individuals. It is a blend of various ethnic groups, with the Igbo as the main ethnic group. The demographic consists of a blend of young families, students, professionals, and retirees. The varied demographic makeup of the city affects the necessity for different healthcare services. Parklane Teaching Hospital, referred to as Enugu State University Teaching Hospital (ESUTH), is a prominent tertiary healthcare facility in southeastern Nigeria. It acts as a referral hub for hospitals and clinics in Enugu and surrounding states. The hospital features specialized departments and units such as Pediatrics, Obstetrics and Gynecology, Surgery, Internal Medicine, and Public Health.

The Pediatric Department at Parklane Teaching Hospital offers extensive healthcare services for children, such as regular check-ups, vaccinations, management of acute and chronic conditions, nutritional guidance, and emergency services. The hospital operates a bustling outpatient clinic, inpatient wards, and specialized departments including the Neonatal Intensive Care Unit (NICU). The economy of Enugu is mainly fueled by services, trade, and a limited industrial sector. The city features multiple educational establishments, including universities that enhance its dynamic economy. In spite of the economic endeavors, notable socio-economic inequalities exist. Certain families encounter difficulties associated with poverty, restricted access to healthcare, and malnutrition, affecting children's health results. Enugu, similar to numerous cities in Nigeria, encounters multiple public health issues. Malnutrition, infectious illnesses (including malaria, pneumonia, and diarrhea), and anemia are common in children younger than five years old. These issues are worsened by factors like insufficient healthcare access, inadequate sanitation, and a lack of public health awareness. Selecting Parklane Teaching Hospital as the study location is purposeful because of its function as a key healthcare provider for a wide-ranging and sizable pediatric community. The hospital's extensive pediatric services and its role as a tertiary referral center render it an optimal site for examining the prevalence of anemia and the nutritional status of children under five. The results of this research are anticipated to offer significant perspectives on the health difficulties encountered by young children in this urban environment and guide public health initiatives focused on enhancing child health results in Enugu and comparable situations.

### Study population

The study population consists of children under the age of 5 who attend Parklane Teaching Hospital. This includes both inpatients and outpatients.

### Sample size determination

The sample size was determined using a single population proportion formula considering the prevalence of anaemia from the study done by Ughasoro *et al*. (2011) which was 49.2%.

*n* = Z^2^p(1-p)/d^2.^

Where d = margin of error between the sample and the population (d = 5%), *n* = sample size, Z = 95% that is confidence interval (1.96), *p* = prevalence which is 49.2% by Ughasoro *et al*. (2011).

(1.96)^2^ × 0.492 (1-0.492)/ (0.05)^2.^

= 384.

### Sampling technique

A consecutive recruitment for the period time was used for all under 5 children who fulfilled the inclusion criteria during the study period.

### Inclusion and exclusion criteria

**Inclusion Criteria:**

- Children aged 0−59 months
- Children whose parents or guardians give informed consent

**Exclusion Criteria:**

- Children with known chronic diseases or conditions affecting haemoglobin levels
- Children whose parents or guardians do not provide consent

## Data collection

1. **Socio-Demographic Information:** Data on age, sex, birth weight, parental education, and socio-economic status are collected through structured questionnaires administered to the parents or caregivers.
2. **Anthropometric Measurements:**
  - **Weight:** Measured using a digital scale. Children are weighed with minimal clothing, and the weight is recorded to the nearest 0.1 kg.
  - **Height/Length:** Measured using a stadiometer or length board for children under 2 years. Height is recorded to the nearest 0.1 cm.

Anthropometric measurements were obtained using standardized procedures appropriate for children under five years. Weight and recumbent length or standing height were measured according to the child's age and recorded using calibrated equipment. The child's age was calculated from the date of birth and date of assessment, while sex was incorporated into the generation and interpretation of age-specific anthropometric indices. Nutritional status was assessed using WHO Child Growth Standards, including weight-for-age Z-score (WAZ), length/height-for-age Z-score (LAZ/HAZ), weight-for-length/height Z-score (WLZ/WHZ), and BMI-for-age Z-score (BAZ). The corresponding Z-scores were generated using the appropriate WHO growth-reference procedures/software. Measurements were reviewed for completeness and biological plausibility before analysis. Implausible values were excluded from the relevant anthropometric analysis, while participants with missing measurements were retained for other analyses for which complete data were available. This standardized approach ensured that anthropometric status was assessed consistently according to age- and sex-specific WHO reference standards. Nutritional status was assessed using complementary WHO anthropometric indicators appropriate for children under five years. Weight-for-height/length was used primarily to assess body proportionality and identify acute or wasting-related nutritional abnormalities, whereas BMI-for-age provides a body mass index adjusted for the child's age and sex and can similarly reflect thinness or excess body weight. Although these indicators are conceptually related because both assess body size relative to stature, they are not identical measures and may produce different classifications for individual children. Their inclusion was therefore intended to provide complementary information on anthropometric status rather than to treat them as completely independent nutritional outcomes. The findings from these indicators were interpreted cautiously, with emphasis on their established WHO definitions and without implying that significant associations between haemoglobin and one anthropometric indicator necessarily applied equally to the other.

### Haemoglobin measurement

#### Collection of blood samples and test procedures

With the help of the Medical Laboratory Scientists in the laboratory sections of the clinics, 2mls or drops of venous or peripheral blood samples were collected from each child after cleaning the sites of collection with 70% alcohol or spirit; some others who were too small to receive children's needles were pricked. The samples that were collected were put into Ethylene diamine tetra-acetic acid (EDTA) containers to avoid clotting and ensure preservation of the samples. The samples were taken to the laboratory for testing. The blood samples were used for packed cell volume and haemoglobin estimation. The haemoglobin estimation was done using Full Blood Count Autoanalyser and children with a haemoglobin concentration less than 11.0 g/dL were considered anaemic.Anaemia is classified based on WHO criteria:

- Mild Anaemia: Haemoglobin level of 10.0−10.9 g/dL
- Moderate Anaemia: Haemoglobin level of 7.0−9.9 g/dL
- Severe Anaemia: Haemoglobin level <7.0 g/dL

Anaemia was defined according to age-specific haemoglobin thresholds appropriate for children under five years. Haemoglobin concentration was measured using the specified laboratory method, and participants were classified as anaemic or non-anaemic based on the applicable reference criteria. Where severity classification was applied, anaemia was categorized according to the corresponding age-specific haemoglobin ranges. Particular attention was given to younger infants because haemoglobin concentrations undergo substantial physiological changes during early infancy; therefore, thresholds applicable to older children were not extrapolated inappropriately to infants below six months.

## Data analysis

Data are entered into a database and analyzed using SPSS software. Descriptive statistics (mean, standard deviation, frequencies, and percentages) are used to summarize the data. The prevalence of anaemia and malnutrition (stunting, wasting, and underweight) are calculated. Chi-square tests are used to assess associations between socio-demographic factors and the prevalence of anaemia and malnutrition. A *p*-value < 0.05 is considered statistically significant.

## Ethical approval

Ethical approval was obtained from National Open University of Nigeria (ETC/2025/04/NOUN232146447). Informed consent is obtained from parents or caregivers of all participating children. All data are anonymized to ensure confidentiality.

## Results

Table 1 indicates a notable statistical correlation between the weight-for-age Z-score of respondents and their haemoglobin levels (anaemia status) (*p* < 0.000), maternal nutrition (*p* < 0.001), socioeconomic status (*p* = 0.000), parental income level (*p* < 0.000), and occupation (*p* < 0.000). Nonetheless, the respondent's weight-for-age Z-score showed no statistically significant correlation (*P* > 0.05) with their packed cell volume (PCV). Table 2 indicates that the height/length for age Z-score of the participants exhibited a statistically significant relationship (*p* < 0.05) with their haemoglobin levels (anaemia status), packed cell volume (PCV), maternal nutrition, socioeconomic status, as well as parental income and occupation. Table 3 illustrates a noteworthy association between the respondent's BMI for age and haemoglobin level (anaemia status) (*p* = 0.013), packed cell volume (*p* < 0.000), maternal nutrition (*p* < 0.002), parental occupation (*p* < 0.032), whereas no significant link was found between their BMI for age and socioeconomic status (*p* > 0.541) or parental income status (*p* > 0.061). No statistically significant relationship (*p* > 0.05) was found between the weight-for-height/length Z-score of the respondents and their haemoglobin levels (anaemia status), socioeconomic status, parental income, or parental occupation. A significant statistical correlation was found between the weight for height/length Z-score of the respondent and their packed cell volume (*p* < 0.000) as well as maternal nutrition (*p* < 0.000) (Table 4). Table 5 reveals a statistically significant relationship between the haemoglobin level of respondents (anaemia status) and their socioeconomic characteristics, including age [*p* < 0.000], gender [*p* = 0.043], parents' education level [*p* < 0.000], income [*p* < 0.000], socioeconomic status [*p* < 0.000], and maternal nutrition [*p* < 0.000].

**Table 1:** Association between haemoglobin level (anaemic), PCV, maternal nutrition, socioeconomic status & weight for age zscore classification of the respondents.

	Weight for Age Zscore				ᵡ^2^	*P*-Value
	Normal	Underweight	Severely Underweight	Total		
Variables	F (%)	F (%)	F (%)	F (%)		
Anaemic Status						
Moderately anaemic	25 (6.5)	37 (9.7)	3 (0.8)	65 (17.0)		
Mildly anaemic	24 (6.3)	89 (23.2)	15 (3.9)	128 (33.4)	90.772	0.000
Normal	132 (72.93)	58 (15.1)	0 (0.0)	191 (49.6)		
Total	181 (47.3)	184 (48.0)	18 (4.7)	384 (100.0)		
Packed Cell Volume						
Low PCV	1 (0.3)	1 (0.3)	1 (0.3)	3 (0.8)		
Normal PCV	189 (47.0)	183 (47.8)	17 (4.4)	381 (99.2)	5.535	0.063
Total	181 (47.3)	184 (48.0)	18 (4.7)	384 (100.0)		
Maternal Nutrition						
Normal	115 (30.0)	132 (34.5)	15 (3.9)	263 (68.4)		
Malnourished	61 (15.9)	45 (11.7)	0 (0.0)	106 (27.7)	17.651	0.001
Underweight	5 (1.3)	7 (1.8)	3 (0.8)	15 (3.9)		
Total	181 (47.3)	184 (48.0)	18 (4.7)	384 (100.0)		
Socioeconomic Status						
Low	65 (17.0)	52 (13.6)	0 (0.0)	117 (30.5)		
Middle	28 (7.3)	105 (27.4)	18 (4.7)	151 (39.4)	105.825	0.000
High	88 (23.0)	27 (7.0)	0 (0.0)	116 (30.0)		
Total	181 (47.3)	184 (48.0)	18 (4.7)	384 (100.0)		
Parental Income Level						
Low	68 (17.8)	55 (14.4)	1 (0.3)	124 (32.4)		
Middle	25 (6.5)	105 (27.4)	17 (4.4)	147 (38.4)	110.813	0.000
High	88 (23.0)	24 (6.3)	0 (0.0)	113 (29.2)		
Total	181 (47.3)	184 (48.0)	18 (4.7)	384 (100.0)		
Parental Occupation						
Teacher	38 (9.9)	2 (0.5)	2 (0.5)	43 (11.0)		
Farmer	23 (6.0)	14 (3.7)	0 (0.0)	37 (9.7)		
Medical Doctor	36 (9.4)	1 (0.3)	0 (0.0)	37 (9.7)		
Nurse	5 (1.3)	33 (8.6)	1 (0.3)	39 (10.2)		
Labourer	38 (9.9)	2 (0.5)	0 (0.0)	40 (10.4)		
Engineer	19 (5.0)	22 (5.7)	0 (0.0)	41 (10.7)		
Shopkeeper	1 (0.3)	32 (8.4)	0 (0.0)	33 (8.6)		
Maid	4 (1.0)	30 (7.8)	0 (0.0)	34 (8.9)	327.028	0.000
Lecturer	15 (3.9)	19 (5.0)	0 (0.0)	34 (8.9)		
Driver	1 (0.3)	18 (4.7)	15 (3.9)	34 (8.9)		
Street Vendor	0 (0.0)	2 (0.5)	0 (0.0)	2 (0.5)		
Shop Owner	1 (0.3)	2 (0.5)	0 (0.0)	3 (0.8)		
Construction Worker	0 (0.0)	3 (0.8)	0 (0.0)	3 (0.8)		
Lawyer	0 (0.0)	3 (0.8)	0 (0.0)	3 (0.8)		
Janitor	0 (0.0)	1 (0.3)	0 (0.0)	1 (0.3)		
Total	181 (47.3)	184 (48.0)	18 (4.7)	384 (100.0)		

**Table 2:** Association between haemoglobin level (anaemic status), packed cell volume (PCV), maternal nutrition, socioeconomic status and the height/length for age zscore classification of the respondents.

	Height/Length for Age Zscore				ᵡ^2^	*P*-Value
	Normal	Stunted	Severely Stunted	Total		
Variables	F (%)	F (%)	F (%)	F (%)		
Anaemic Status						
Moderately anaemic	4 (1.0)	33 (8.6)	28 (7.3)	65 (17.0)		
Mildly anaemic	3 (0.8)	86 (22.5)	39 (10.2)	128 (33.4)	132.533	0.000
Normal	93 (24.3)	90 (23.5)	7 (1.8)	191 (49.6)		
Total	100 (26.1)	209 (54.6)	74 (19.3)	384 (100.0)		
Packed Cell Volume						
Low PCV	0 (0.0)	0 (0.0)	3 (0.8)	3 (0.8)		
Normal PCV	100 (26.1)	209 (54.6)	71 (18.5)	381 (99.2)	12.626	0.002
Total	100 (26.1)	209 (54.6)	74 (19.3)	384 (100.0)		
Maternal Nutrition						
Normal	44 (11.5)	156 (40.7)	62 (16.2)	262 68.4)		
Malnourished	55 (14.4)	50 (13.1)	106 (27.7)	106 (27.7)	87.127	0.000
Underweight	1 (0.3)	3 (0.8)	15 (3.9)	16 (3.9)		
Total	100 (26.1)	209 (54.6)	74 (19.3)	384 (100.0)		
Socioeconomic Status						
Low	55 (14.4)	53 (13.8)	9 (2.3)	117 (30.5)		
Middle	6 (1.6)	87 (22.7)	58 (15.1)	151 (39.4)	100.406	0.000
High	39 (10.2)	69 (18.0)	7 (1.8)	116 (30.0)		
Total	100 (26.1)	209 (54.6)	74 (19.3)	384 (100.01)		
Parental Income Level						
Low	55 (14.4)	55 (14.4)	14 (3.7)	124 (32.4)		
Middle	6 (1.6)	86 (22.5)	55 (14.4)	147 (38.4)	91.789	0.000
High	39 (10.2)	68 (17.8)	5 (1.3)	113 (29.2)		
Total	100 (26.1)	209 (54.6)	74 (19.3)	384 (100.0)		
Parental Occupation						
Teacher	3 (0.8)	36 (9.4)	3 (0.8)	43 (11.0)		
Farmer	1 (0.3)	33 (8.6)	3 (0.8)	37 (9.7)3		
Medical Doctor	34 (8.9)	1 (0.3)	2 (0.5)	37 (9.7)		
Nurse	2 (0.5)	20 (5.2)	17 (4.4)	39 (10.2)		
Labourer	34 (8.9)	5 (1.3)	1 (0.3)	40 (10.4)		
Engineer	1 (0.3)	36 (9.4)	4 (1.0)	41 (10.7)		
Shopkeeper	1 (0.3)	31 (8.1)	1 (0.3)	33 (8.6)	450.586	0.000
Maid	20 (5.2)	14 (3.7)	0 (0.0)	34 (8.9)		
Lecturer	1 (0.3)	32 (8.4)	1 (0.3)	34 (8.9)		
Driver	1 (0.3)	0 (0.0)	33 (8.6)	34 (8.9)		
Street Vendor	0 (0.0)	0 (0.0)	2 (0.5)	2 (0.5)		
Shop Owner	1 (0.3)	0 (0.0)	2 (0.5)	3 (0.8)		
Construction Worker	0 (0.0)	1 (0.3)	2 (0.5)	3 (0.8)		
Lawyer	1 (0.3)	0 (0.0)	2 (0.5)	3 (0.8)		
Janitor	0 (0.0)	0 (0.0)	1 (0.3)	1 (0.3)		
Total	100 (26.1)	209 (54.6)	74 (19.3)	384 (100.0)		

**Table 3:** Association between haemoglobin level (anaemic status), packed cell volume (PCV), maternal nutrition, socioeconomic status and the BMI for age zscore classification of the respondents.

	BMI for Age Zscore				ᵡ^2^	*P*-Value
	Overweight	Normal	Wasted	Total		
Variables	F (%)	F (%)	F (%)	F (%)		
Anaemic Status						
Moderately anaemic	4 (1.0)	61 (15.9)	0 (0.0)	65 (17.0)		
Mildly anaemic	1 (0.3)	126 (32.9)	1 (0.3)	128 (33.4)	12.697	0.013
Normal	1 (0.3)	189 (49.3)	0 (0.0)	191 (49.6)		
Total	6 (1.6)	376 (98.2)	1 (0.3)	384 (100.0)		
Packed Cell Volume						
Low PCV	1 (0.3)	2 (0.5)	0 (0.0)	3 (0.8)		
Normal PCV	5 (1.3)	374 (97.7)	1 (0.3)	381 (99.2)	19.791	0.000
Total	6 (1.6)	376 (98.2)	1 (0.3)	384 (100.0)		
Maternal Nutrition						
Normal	3 (0.8)	259 (67.6)	0 (0.0)	262 (68.4)		
Malnourished	1 (0.3)	104 (27.2)	1 (0.3)	106 (27.7)	16.645	0.002
Underweight	2 (0.5)	13 (3.4)	0 (0.0)	16 (3.9)		
Total	6 (1.6)	376 (98.2)	1 (0.3)	384 (100.0)		
Socioeconomic Status						
Low	3 (0.3)	114 (29.8)	0 (0.0)	117 (30.5)		
Middle	1 (0.3)	149 (38.9)	1 (0.3)	151 (39.4)	3.100	0.541
High	2 (0.5)	113 (29.5)	0 (0.0)	116 (30.0)		
Total	6 (1.6)	376 (98.2)	1 (0.3)	384 (100.0)		
Parental Income Level						
Low	5 (1.3)	119 (31.1)	0 (0.0)	124 (32.4)		
Middle	1 (0.3)	145 (37.9)	1 (0.3)	147 (38.4)	9.012	0.061
High	0 (0.0)	112 (29.2)	0 (0.0)	113 (29.2)		
Total	6 (1.6)	376 (98.2)	1 (0.3)	384 (100.0)		
Parental Occupation						
Teacher	1 (0.3)	41 (10.7)	0 (0.0)	43 (11.0)		
Farmer	3 (0.8)	34 (8.9)	0 (0.0)	37 (9.7)		
Medical Doctor	1 (0.3)	36 (9.4)	0 (0.0)	37 (9.7)		
Nurse	0 (0.0)	38 (9.9)	1 (0.3)	39 (10.2)		
Labourer	0 (0.0)	40 (10.4)	0 (0.0)	40 (10.4)		
Engineer	0 (0.0)	41 (10.7)	0 (0.0)	41 (10.7)		
Shopkeeper	0 (0.0)	33 (8.6)	0 (0.0)	33 (8.6)	43.405	0.032
Maid	0 (0.0)	34 (8.9)	0 (0.0)	34 (8.9)		
Lecturer	0 (0.0)	34 (8.9)	0 (0.0)	34 (8.9)		
Driver	0 (0.0)	34 (8.9)	0 (0.0)	34 (8.9)		
Street Vendor	0 (0.0)	2 (0.5)	0 (0.0)	2 (0.5)		
Shop Owner	0 (0.0)	3 (0.8)	0 (0.0)	3 (0.8)		
Construction Worker	0 (0.0)	3 (0.8)	0 (0.0)	3 (0.8)		
Lawyer	1 (0.3)	2 (0.5)	0 (0.0)	3 (0.8)		
Janitor	0 (0.0)	1 (0.3)	0 (0.0)	1 (0.3)		
Total	0 (0.0)	376 (98.2)	1 (0.3)	384 (100.0)		

**Table 4:** Association between haemoglobin level (anaemic status), packed cell volume (PCV), maternal nutrition, socioeconomic status and the weight for height/length zscore classification of the respondents.

	Weight for Height/Length Zscore				ᵡ^2^	*P*-Value
	Overweight	Normal	Wasted	Total		
Variables	F (%)	F (%)	F (%)	F (%)		
Anaemic Status						
Moderately anaemic	1 (0.3)	64 (16.7)	0 (0.0)	65 (17.0)		
Mildly anaemic	0 (0.0)	127 (33.2)	1 (0.3)	128 (33.4)	6.897	0.141
Normal	0 (0.0)	190 (49.6)	0 (0.0)	191 (49.6)		
Total	1 (0.3)	381 (99.5)	1 (0.0)	384 (100.0)		
Packed Cell Volume						
Low PCV	1 (0.3)	2 (0.5)	0 (0.0)	3 (0.8)		
Normal PCV	0 (0.0)	379 (99.0)	1 (0.3)	381 (99.2)	127.002	0.000
Total	1 (0.3)	381 (99.5)	1 (0.3)	384 (100.0)		
Maternal Nutrition						
Normal	0 (0.0)	262 (68.4)	0 (0.0)	262 (68.4)		
Malnourished	0 (0.0)	105 (27.4)	1 (0.3)	106 (27.7)	27.213	0.000
Underweight	1 (0.3)	14 (3.7)	0 (0.0)	16 (3.9)		
Total	1 (0.3)	381 (99.5)	1 (0.3)	384 (100.0)		
Socioeconomic Status						
Low	1 (0.3)	116 (30.3)	0 (0.0)	117 (30.5)		
Middle	0 (0.0)	150 (39.2)	1 (0.3)	151 (39.4)	3.815	0.432
High	0 (0.0)	115 (30.0)	0 (0.0)	116 (30.0)		
Total	1 (0.3)	381 (99.5)	1 (0.3)	384 (100.0)		
Parental Income Level						
Low	0 (0.0)	124 (32.4)	0 (0.0)	124 (32.4)		
Middle	1 (0.3)	145 (37.9)	1 (0.3)	147 (38.4)	3.2288	0.520
High	0 (0.0)	112 (29.2)	0 (0.0)	113 (29.2)		
Total	1 (0.3)	381 (99.5)	1 (0.3)1	384 (99.5)		
Parental Occupation						
Teacher	0 (0.0)	42 (11.0)	0 (0.0)	43 (11.0)		
Farmer	1 (0.3)	36 (9.4)	0 (0.0)	37 (9.7)		
Medical Doctor	0 (0.0)	37 (9.7)	0 (0.0)	37 (9.7)		
Nurse	0 (0.0)	38 (9.9)	1 (0.3)	39 (10.2)		
Labourer	0 (0.0)	40 (10.4)	0 (0.0)	40 (10.4)		
Engineer	0 (0.0)	41 (10.7)	0 (0.0)	41 (10.7)		
Shopkeeper	0 (0.0)	33 (8.6)	0 (0.0)	33 (8.6)		
Maid	0 (0.0)	34 (8.9)	0 (0.0)	34 (8.9)	18.214	0.921
Lecturer	0 (0.0)	34 (8.9)	0 (0.0)	34 (8.9)		
Driver	0 (0.0)	34 (8.9)	0 (0.0)	34 (8.9)		
Street Vendor	0 (0.0)	2 (0.5)	0 (0.0)	2 (0.5)		
Shop Owner	0 (0.0)	3 (0.8)	0 (0.0)	3 (0.8)		
Construction Worker	0 (0.0)	3 (0.8)	0 (0.0)	3 (0.8)		
Lawyer	0 (0.0)	3 (0.8)	0 (0.0)	3 (0.8)		
Janitor	0 (0.0)	1 (0.3)	0 (0.0)	1 (0.3)		
Total	1 (0.3)	381 (0.3)	1 (0.3)	384 (100.0)		

**Table 5:** Association between the socioeconomic characteristics and haemoglobin level (anaemic status) of the respondents.

	Haemoglobin Level (Anaemic Status)				ᵡ^2^	*P*-value
	Moderately Anaemic	Mildly Anaemic	Normal	Total		
Variable	F (%)	F (%)	F (%)	F (%)		
Age Group						
6−12 months	1 (0.3)	1 (0.3)	84 (21.9)	87 (22.5)		
13–24 months	8 (2.1)	73 (19.1)	38 (9.9)	119 (31.1)		
25–36 months	39 (10.2)	38 (9.9)	57 (14.9)	134 (35.0)	158.820	0.000
37–48 months	17 (4.4)	16 (4.2)	9 (2.3)	43 (11.0)		
49–54 months	0 (0.0)	0 (0.0)	2 (0.5)	2 (0.5)		
55–59 Months	0 (0.0)	0 (0.0)	2 (0.5)	2 (0.5)	158.820	<0.001
Total	65 (17.0)	128 (33.4)	190 (49.6)	384 (100.0)		
Gender						
Male	37 (9.7)	72 (18.8)	83 (21.7)	193 (50.1)		
Female	28 (7.3)	56 (14.6)	107 (27.9)	191 (49.9)	6.275	0.043
Total	65 (17.0)	128 (33.4)	190 (49.6)	384 (100.0)		
Parent Educational Level						
None	2 (0.5)	2 (0.5)	72 (18.8)	76 (19.8)		
Primary Education	33 (8.6)	1 (0.3)	4 (1.0)	39 (9.9)		
Secondary Education	22 (5.7)	47 (12.3)	1 (0.3)	70 (18.3)		
Diploma	2 (0.5)	40 (10.4)	8 (2.1)	50 (13.1)	449.957	0.000
Bachelor's	4 (1.0)	3 (0.8)	69 (18.0)	76 (19.8)		
Master's	2 (0.5)	35 (9.1)	2 (0.5)	39 (10.2)		
Doctorate	0 (0.0)	0 (0.0)	34 (8.9)	34 (8.9)		
Total	65 (17.0)	128 (33.4)	190 (49.6)	384 (100.0)		
Parental Income Level						
Low	37 (9.7)	7 (1.8)	80 (20.9)	124 (32.4)		
Middle	23 (6.0)	118 (30.8)	6 (1.6)	147 (38.4)	288.621	0.000
High	5 (1.3)	3 (0.8)	104 (27.2)	113 (29.2)		
Total	65 (17.0)	128 (33.4)	190 (49.6)	384 (100.0)		
Socioeconomic Status						
Low	37 (9.7)	4 (1.0)	76 (19.8)	117 (30.5)		
Middle	23 (6.0)	119 (31.0)	9 (2.3)	151 (39.4)		
High	5 (1.3)	5 (1.3)	105 (27.4)	116 (30.0)	282.389	0.000
Total	65 (17.0)	128 (33.4)	190 (49.6)	384 (100.0)		
Maternal Nutrition						
Normal	24 (6.3)	122 (31.9)	116 (30.3)	262 (68.4)		
Malnourished	35 (9.1)	2 (0.5)	69 (18.0)	106 (27.7)	82.906	0.000
Underweight	6 (1.6)	4 (1.0)	5 (1.3)	16 (3.9)		
Total	65 (17.0)	128 (33.4)	190 (49.6)	384 (100.0)		

## Discussion

The research identified a statistically significant link between the weight-for-age Z-score of the respondents and various factors, such as haemoglobin level (*p* < 0.000), maternal nutrition (*p* < 0.001), socioeconomic status (*p* < 0.000), parental income level (*p* < 0.000), and occupation (*p* < 0.000). These results correspond with current research that highlights the importance of maternal nutrition and socioeconomic influences on children's nutritional health and overall well-being. For example, research by Ruel and Alderman (8) emphasized that inadequate maternal nutrition and low socioeconomic status directly affect child growth and development, leading to stunted growth and a higher risk of anaemia. Notably, although weight for age Z-score was significantly linked to anaemia status, no significant connection with PCV was found (*p* > 0.05). This may imply that although weight for age can indicate nutritional status, it does not always align with the body's erythropoietic response as assessed by PCV, a notion backed by additional studies showing that haemoglobin and PCV can occasionally differ according to hydration levels and various physiological factors (9). The research indicates a notable correlation between the height/length for age Z-score and several factors, such as haemoglobin level (*p* < 0.05) and maternal nutrition. This discovery supports research such as that by Victora et al. (10), which indicated that childhood anaemia is frequently associated with inadequate growth, underscoring the necessity of tackling nutritional deficiencies during vital growth phases. Maternal nutrition has a significant impact, as insufficient nutrition can result in anaemia and stunted growth in children.

The relationship between BMI for age and haemoglobin level (*p* < 0.013), PCV (*p* < 0.000), maternal nutrition (*p* < 0.002), and parental occupation (*p* < 0.032) strengthens the idea that nutritional intake and socioeconomic factors play a crucial role in children's health outcomes. The absence of a notable link with socioeconomic status (*p* > 0.541) and parental income status (*p* > 0.061) may indicate that although these factors influence nutritional quality, they do not directly relate to BMI in this case, possibly due to differences in diet quality and healthcare access. It is noteworthy that there are no significant associations (*p* > 0.05) between the weight for height/length Z-score and haemoglobin levels, socioeconomic status, parental income, and parental occupation. The significant link with maternal nutrition (*p* < 0.000) and PCV (*p* < 0.000) indicates that, although overall socioeconomic status might not directly influence immediate nutritional indicators, maternal nutrition is still a vital factor for both weight and general health in children. In Enugu State, 49.6% of respondents exhibited normal haemoglobin levels, while 33.4% were identified as mildly anaemic and 17.0% as moderately anaemic. The significant occurrence of anaemia among participants is alarming, particularly since almost all (98.2%) exhibited normal PCV. This difference may suggest that although PCV levels are typically normal, there are common nutritional deficiencies impacting haemoglobin production. This aligns with results from research such as that conducted by Balarajan et al. (11), which discovered a significant prevalence of anaemia in groups with typically normal iron reserves.

The research identified notable links between hemoglobin levels and various socioeconomic factors, with *p*-values indicating robust correlations: Younger individuals or specific age brackets may face increased anemia rates owing to elements like growth requirements or nutritional deficiencies. A study conducted by Mainasara et al. (11) revealed that adolescents experienced a greater prevalence of anemia, largely due to heightened nutritional requirements during periods of growth (12). The variation in hemoglobin levels between men and women is frequently associated with physiological differences, such as menstruation in women, which may lead to anemia. A systematic review by Geta et al. (13) highlighted that women are at a greater risk of anemia than men, especially in developing nations where nutritional deficiencies are common (12). Increased educational levels of parents frequently correlate with improved health outcomes for their children, since educated parents generally possess superior understanding of nutrition and healthcare. A study by Fadare et al. (13) found that children whose parents have higher levels of education exhibited notably elevated hemoglobin levels, highlighting the impact of parental education on children's health (14). Income levels are directly connected to the capacity to obtain nutritious foods and health care services. Research by Okafor et al. (15) showed that families with lower incomes tend to face food insecurity, resulting in increased anemia rates among their children (15). A thorough assessment of a person's or household's financial and social standing can significantly influence their nutritional condition. A study conducted by Owoaje et al. (16) revealed that lower socioeconomic status was strongly linked to worse health outcomes, such as higher anemia rates (16). Maternal nutrition throughout pregnancy greatly impacts the hemoglobin levels in children. A study by Adinma et al. (17) indicated that inadequate maternal nutrition was a significant risk factor for anemia in children, emphasizing the importance of maternal health in preventing anemia (17).

The findings of this study demonstrate an important relationship between haemoglobin status and anthropometric characteristics among children under five years receiving healthcare services at Parklane Teaching Hospital, Enugu. The observed prevalence of anaemia highlights the continued importance of routine haemoglobin assessment in young children, particularly in healthcare settings where nutritional and infectious conditions may coexist. The observed anthropometric abnormalities further indicate that impaired nutritional status may occur alongside anaemia, although the cross-sectional design does not permit determination of the temporal direction of this relationship. The relationship between haemoglobin concentration and anthropometric status may reflect the complex interaction between nutritional adequacy, growth, infection, inflammation, and other determinants of child health. However, these mechanisms were not directly evaluated in the present study. In particular, iron status, dietary iron intake, inflammatory biomarkers, parasitic infections, and other specific causes of anaemia were not comprehensively assessed. Therefore, these factors should be regarded as possible explanations rather than established causes of the observed associations (18). The findings are broadly considered in relation to previous studies among children under five years in Nigeria and other African settings. Differences between studies may reflect variations in study populations, geographical settings, socioeconomic circumstances, disease burden, dietary practices, healthcare access, anthropometric assessment methods, and definitions of anaemia. Such contextual differences should be considered when interpreting comparisons across studies. The socioeconomic findings also suggest that household and parental characteristics may be relevant to childhood haemoglobin and nutritional status. Nevertheless, the observed relationships should not be interpreted as independent effects unless supported by adjusted analyses. Socioeconomic status, parental education, household resources, dietary practices, and healthcare utilization are interrelated factors, and residual confounding may therefore remain (19, 20, 21).

### Limitations of the study

This study has several limitations that should be considered when interpreting the findings. First, its cross-sectional design permits assessment of associations but does not establish temporal or causal relationships between haemoglobin status and anthropometric characteristics. Second, the study was conducted at a single tertiary teaching hospital, and the use of consecutive recruitment may have introduced selection or referral bias because children attending a tertiary facility may differ from the general population of children under five years in Enugu. Consequently, the findings may not be directly generalizable to all children in the wider community. Third, information on some socioeconomic and maternal characteristics was obtained from caregivers and may therefore be subject to recall or reporting bias. In addition, maternal nutritional status was not assessed comprehensively using dietary intake data or biochemical nutritional biomarkers. Fourth, although haemoglobin concentration was assessed, the study did not comprehensively investigate the specific causes of anaemia, such as iron deficiency, malaria, parasitic infections, inflammation, haemoglobinopathies, or other micronutrient deficiencies. Therefore, the observed association between haemoglobin status and anthropometric characteristics should not be interpreted as evidence of a specific underlying mechanism. Finally, some subgroup categories contained relatively small numbers of participants, which may have reduced statistical power and affected the precision of some estimates. Although relevant demographic, socioeconomic, and anthropometric variables were considered in the analysis, residual confounding cannot be completely excluded. Future multicentre longitudinal studies involving larger and more representative populations, detailed dietary assessment, biochemical measures of iron and micronutrient status, infection and inflammation markers, and other relevant determinants are recommended to provide a more comprehensive understanding of the relationship between anaemia and child growth and nutritional status.

## Conclusion

This research shows a notable link between haemoglobin levels and various anthropometric measurements in children under five years old visiting Parklane Teaching Hospital, Enugu. Weight-for-age, height/length-for-age, and BMI-for-age were strongly associated with anaemia status, underscoring the complex connection between growth patterns and blood health. Maternal nutrition and socioeconomic variables—including education level, income, and job status of parents—were significantly linked to haemoglobin levels, highlighting the complex factors that contribute to childhood anaemia. The results highlight the essential requirement for comprehensive child health programs that simultaneously tackle nutritional shortages, economic inequalities, and maternal wellness. Regular monitoring of anthropometric measurements and haemoglobin levels should be integrated into pediatric health services to identify at-risk children promptly. Focused nutritional interventions, maternal training efforts, and community-oriented economic assistance can together alleviate the challenges of anaemia and growth delays in this at-risk group.

## Data Availability

The raw data supporting the conclusions of this article will be made available by the authors, without undue reservation.
